# Associations between handgrip strength and skeletal muscle mass with all‐cause mortality and cardiovascular mortality in people with type 2 diabetes: A prospective cohort study of the UK Biobank

**DOI:** 10.1111/1753-0407.13464

**Published:** 2023-08-22

**Authors:** Lingqi Wei, Jingjing Zeng, Menglin Fan, Bo Chen, Xiaying Li, Ying Li, Shaoyong Xu

**Affiliations:** ^1^ College of Medicine, Wuhan University of Science & Technology Wuhan China; ^2^ Department of Endocrinology Xiangyang Central Hospital, Affiliated Hospital of Hubei University of Arts and Science Xiangyang China; ^3^ Center for Clinical Evidence‐Based and Translational Medicine Xiangyang Central Hospital, Affiliated Hospital of Hubei University of Arts and Science Xiangyang China

**Keywords:** all‐cause mortality, cardiovascular mortality, handgrip strength, sarcopenia, skeletal muscle mass

## Abstract

**Aims:**

To explore the associations between handgrip strength (HGS) and skeletal muscle mass (SMM) with all‐cause and cardiovascular disease (CVD) mortality risk in type 2 diabetes (T2DM) patients.

**Materials and Methods:**

Data were obtained from the UK Biobank. Baseline survey was conducted between 2006 and 2010, and followed up for a median of 12.52 years. HGS was measured using dynamometer, and SMM was measured using bioelectrical impedance method. Mortality was available via links to the National Health Service Information Centre. Sex‐specific analyses were conducted.

**Results:**

A total of 13 392 T2DM participants were included, with a mean age of 60.39 years and 52.35% men. During the follow‐up, there were 3006 (22.45%) deaths, including 746 (5.57%) CVD deaths. The risk for all‐cause mortality and CVD mortality among both men and women increased progressively with decreasing HGS quartiles (*p* trend <.05). A 1 SD decrease in HGS was found to both increase the all‐cause risk (HR: 1.31 [95% CI: 1.24–1.38]) and CVD mortality risk (HR: 1.35 [95% CI: 1.22–1.50]) for men, and all‐cause risk (HR: 1.26 [95% CI: 1.11–1.42]) and CVD mortality risk (HR: 1.43 [95% CI: 1.09–1.89]) for women. There was no statistically significant trend association between SMM/height^2^ and mortality risk, and the restricted cubic regression splines indicated that SMM/height^2^ showed a U‐shaped nonlinear relationship (*p*_nonlinear_ <.05).

**Conclusions:**

Grip strength displayed a linear downward trend with mortality risk among T2DM patients, whereas muscle mass showed a U‐shaped relationship. Low grip strength seemed to be a better predictor for mortality compared to low muscle mass.

## Graphical abstract

**Highlights**

- The prevalence of low handgrip strength and skeletal muscle mass was higher in women and in patients with long‐duration diabetes and increased with increasing age and decreasing body mass index.
- All‐cause mortality and cardiovascular mortality risk showed a linear decreasing trend with increasing handgrip strength, whereas skeletal muscle mass showed a U‐shaped nonlinear relationship with all‐cause mortality and cardiovascular mortality risk.
- Low handgrip strength was a better predictor of all‐cause mortality and cardiovascular mortality risk than low skeletal muscle mass.

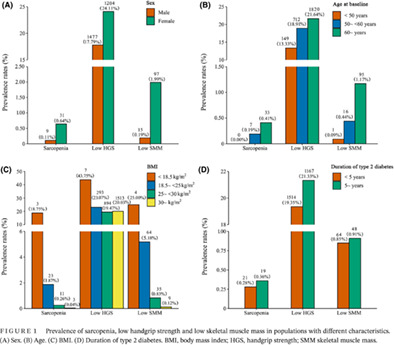

## 1 INTRODUCTION

Sarcopenia is a geriatric syndrome with age‐related loss of muscle mass, decreased muscle strength, and/or decreased somatic function.^1^ In recent years, sarcopenia has received increasing attention. Several previous studies have shown that sarcopenia is strongly associated with a wide range of adverse health outcomes, including fractures, falls, frailty, hospitalization, physical disability, cardiovascular disease (CVD), and even mortality.^2^, ^3^, ^4^, ^5^, ^6^ It not only severely reduces the quality of life of patients but also imposes a high economic burden on individuals and society. Previous studies have shown that sarcopenia can significantly increase the risk of mortality in the general population, particularly in older subjects.^6^ Brown et al showed that sarcopenia is associated with an increased risk of CVD mortality, but with sex specificity, with a 61% increased risk of CVD mortality in women but not in men.^5^

As one of the three most frequent chronic diseases globally, patients with type 2 diabetes usually display a progressive decline in both muscle mass and strength. The effects of sarcopenia in older patients with type 2 diabetes are of increasing concern, particularly with the aging global population. Studies have shown that patients with diabetes are at a higher risk for sarcopenia and have significantly lower muscle mass and strength compared with the general population.^7^, ^8^, ^9^, ^10^ Additionally, patients with diabetes are at a higher risk for all‐cause and CVD mortality compared with the general population. The impact of sarcopenia on the risk for all‐cause and CVD mortality in diabetic patients is of interest. There is growing evidence that sarcopenia and low muscle mass and strength are associated with higher all‐cause and CVD mortality in patients with diabetes mellitus,^11^, ^12^, ^13^, ^14^, ^15^, ^16^ but the relationship is inconsistent with respect to sex specificity. Although both muscle strength and mass are indicators of sarcopenia, each reflects different stages and degrees of physical frailty. Previous studies examining the relationship between muscle strength and mass simultaneously with all‐cause and CVD mortality in diabetic populations are very limited, and previous studies have only used categorical data to investigate this association, limiting our understanding of the true pattern of the association.

In the present study, we used data from the UK Biobank, a large population‐based prospective cohort, to investigate the strength and pattern of associations between sarcopenia, muscle strength, and muscle mass with all‐cause and CVD mortality risk in a diabetic population.

## 2 METHODS

This study is reported in accordance with the Strengthening the Reporting of Observational Studies in Epidemiology (STROBE) guidelines (Table S1).

### 2.1 Study design and population

Data for the study were obtained from the UK Biobank, a large, population‐based prospective cohort study. Between 2006 and 2010, over 500 000 participants aged 37–73 years were recruited.^17^ Baseline assessments included a touchscreen questionnaire, physical measurement, and the collection of biological samples.^18^ The UK Biobank received ethical approval from the North‐West Multi‐center Research Ethics Committee. All participants provided written informed consent. The present study was performed under application number 92014.

Diabetes diagnosis was derived from self‐reported physician diagnosis or medical records. There were 13 531 participants with type 2 diabetes at the baseline survey. In total, 139 individuals with neither grip strength nor muscle mass data were excluded. Of the 13 392 individuals included in the final analysis, 97 were lacking grip strength and 580 were lacking muscle mass data (Figure **S1**).

### 2.2 Exposure measurement

Handgrip strength (HGS) and body composition of participants were determined at baseline. HGS was measured using a Jamar J00105 hydraulic hand dynamometer; the measurement method was previously described in detail.^13^ The mean values of the left and right HGS were expressed in absolute units (kg).^13^ The participants' body weight and total body composition were determined using a bioelectrical impedance method (Tanita BC418MA, Tokyo, Japan). Skeletal muscle mass (SMM) was calculated according to the Janssen equation.^19^ Considering the influence of individual body size, SMM was expressed relative to height squared (SMM/height^2^, kg/m^2^), which was used for subsequent classification and analyses.

According to the European Working Sarcopenia in Older People 2019 (EWGSOP2) recommendations, the cutoff values for low HGS are defined as <27 kg for men and < 16 kg for women; the cutoff values for low SMM are defined as SMM/height^2^ < 7.0 kg/m^2^ for men and <5.5 kg/m^2^ for women.^20^ Self‐reported walking speed in the UK Biobank was used to reflect gait speed to classify the severity of sarcopenia.^21^ Using HGS, SMM/height^2^, and gait speed, according to the EWGSOP2, sarcopenia was classified as nonsarcopenia, defined as HGS in the normal range; presarcopenia, defined as low HGS only; sarcopenia, defined as low HGS and low SMM (gait speed is normal); and severe sarcopenia, defined as sarcopenia with slow gait speed.^20^ However, because the numbers of both, sarcopenia and severe sarcopenia were low (17 and 23, respectively), these two were combined (hereafter referred to as sarcopenia). In addition, we defined sarcopenia in our sensitivity analysis according to the criteria developed by the Foundation for the National Institutes of Health (FNIH) Sarcopenia Project, where the cutoff values for low HGS are defined as <26 kg for men and < 16 kg for women; for low SMM are defined as SMM/body mass index (BMI) <0.789 for men and <0.512 for women.^22^

### 2.3 Outcomes

Mortality data are available via links to the National Health Service Information Centre of England and Wales or the National Health Service Central Register and National Records of Scotland. CVD‐specific mortality was recorded in accordance with the 10th revision of International Classification of Diseases definitions, codes: I20‐I25, I46, I50, and I60‐I64. Participants were censored at the date of available mortality data (October 31, 2021), death, or lost to follow‐up, whichever came first.

### 2.4 Covariates

A touchscreen questionnaire was used to investigate sociodemographic characteristics at the baseline assessment. Smoking status was categorized as never, previous, and current smoker. Alcohol drinking was categorized as never, previous, occasionally (1–3 times/month or special occasions only), weekly (1–4 times/week), and daily (daily/almost daily). Self‐reported physical activity was obtained through the International Physical Activity Questionnaire short form.^23^ The total physical activity was calculated and expressed as metabolic equivalents (MET‐h/week). Physical activity is an important factor in causing changes in skeletal muscle and HGS.^24^ Previous meta‐analyses have shown that the level of physical activity is an important factor in influencing the risk of mortality.^25^ Sleep chronotype was classified as morning person and evening person by a simple question “Do you consider yourself to be”. A questionnaire was used to obtain patients' recent insulin medication history. Dietary information was obtained by a touchscreen questionnaire on self‐reported frequency of consumption of food groups, including red meat, processed meat, oily fish, fruits, and vegetables.

### 2.5 Statistical analyses

Analyses were conducted using SAS 9.4 software (SAS Institute, Cary, NC, USA) and R (4.2.1), and a *p* value <.05 was considered to be statistically significant. All statistical analyses were performed according to sex classification. Continuous variables were expressed as mean ± SD, and categorical variables were expressed as frequencies and percentages. Analysis of variance or chi‐square test was used to compare the characteristics of the populations with different sarcopenia classifications. The distribution of sarcopenia, low HGS, and low SMM in populations were also compared with different characteristics. Pearson correlation analysis was used to explore the correlation between HGS and SMM/height^2^.

The association of sarcopenia, HGS, and SMM with all‐cause mortality and CVD mortality was investigated using a Cox proportional risk model, and hazard ratios (HRs) and 95% confidence intervals (CIs) were calculated. First, sarcopenia, low HGS, and low SMM were defined according to the EWGSOP2 criteria, and the effect on the risk of mortality was assessed. Subsequently, HRs were performed for age‐specific quartiles of HGS and SMM/height^2^ using the highest quartile (Q4) as the reference category, and a trend test was performed. In addition, HGS and SMM/height^2^ were used as continuous variables to calculate the HRs per 1 SD reduction. To investigate the nonlinear association between HGS and SMM/height^2^ with the risk of mortality, the multivariable restricted cubic regression splines with four knots was conducted. To check overall statistical significance and the nonlinearity of the exposures, likelihood ratio tests were used to estimate nonlinearity *p* values, with a *p* value <.05 suggesting a nonlinear relationship between the exposure and mortality risk.^26^

Because of the small number of subjects classified into the sarcopenia and low SMM groups, stratified analyses of the relationship between low HGS and mortality risk were performed only by age, BMI, and duration of diabetes mellitus. The sarcopenia population was also reclassified according to the FNIH criteria to investigate whether there were differences in the relationship between sarcopenia and mortality risk for different criteria classifications. To minimize the effect of reverse causality on the study results, a landmark analysis in the sensitivity analysis was performed by excluding those who had an event within the first 2 years of follow‐up. Due to the collinearity of BMI and fat mass (correlation coefficient: 0.92; *p* < .001), we included only one of them as a confounder. In addition, we conducted a sensitivity analysis to further explore SMM/height^2^ quartiles with fat mass as a confounder.

## 3 RESULTS

Of the 13 392 participants included in the analysis, with a mean follow‐up of 12.52 ± 3.09 years, there were 3006 (22.45%) deaths, including 746 (5.57%) CVD deaths. The mean age of the participants was 60.39 ± 6.76 years and the mean BMI was 31.87 ± 5.96 kg/m^2^. There were 12 716 individuals available for the sarcopenia classification, of whom 7901 (62.13%) were men, and the baseline characteristics of participants by sarcopenia category and sex are presented in Table 1.

**TABLE 1:** Baseline characteristics by categories of sarcopenia and sex.

Characteristics	Total population (*N* = 13 392)	Men				Women			
		Nonsarcopenia (*N* = 6537)	Presarcopenia (*N* = 1355)	Sarcopenia (*N* = 9)	*p*	Nonsarcopenia (*N* = 3674)	Presarcopenia (*N* = 1110)	Sarcopenia (*N* = 31)	*p*
Age (years)	60.39 ± 6.76	60.47 ± 6.68	61.69 ± 6.41	65.22 ± 3.56	<.001	59.54 ± 7.09	60.84 ± 6.27	62.94 ± 5.12	<.001
BMI (kg/m^2^)	31.87 ± 5.96	31.30 ± 5.28	30.85 ± 5.63	23.32 ± 3.87	<.001	32.74 ± 6.61	33.28 ± 6.53	24.03 ± 3.93	<.001
BMI category					<.001				<.001
< 18.5 kg/m^2^	16 (0.12)	6 (0.09)	4 (0.30)	1 (11.11)		3 (0.08)	0	2 (6.45)	
18.5–24.9 kg/m^2^	1277 (9.54)	572 (8.75)	175 (12.92)	5 (55.56)		376 (10.23)	82 (7.39)	18 (58.06)	
25–29.9 kg/m^2^	4367 (32.61)	2393 (36.61)	486 (35.87)	2 (22.22)		1003 (27.30)	304 (27.39)	9 (29.03)	
≥ 30 kg/m^2^	7732 (57.74)	3566 (54.55)	690 (50.92)	1 (11.11)		2292 (62.38)	724 (65.23)	2 (6.45)	
Ethnicity					<.001				<.001
White	11 335 (84.64)	5732 (87.69)	1043 (76.97)	5 (55.56)		3090 (84.10)	862 (77.66)	19 (61.29)	
Asian or Asian British	879 (6.56)	346 (5.29)	189 (13.95)	4 (44.44)		183 (4.98)	110 (9.91)	11 (35.48)	
Black or Black British	380 (2.84)	131 (2.00)	26 (1.92)	0		163 (4.44)	46 (4.14)	0	
Other	798 (5.96)	328 (5.02)	97 (7.16)	0		238 (6.48)	92 (8.29)	1 (3.23)	
Professional qualifications					<.001				<.001
College or university	2783 (20.78)	1485 (22.72)	268 (19.78)	2 (22.22)		734 (19.98)	176 (15.86)	6 (19.35)	
A levels/AS levels or equivalent	1175 (8.77)	607 (9.29)	87 (6.42)	0		337 (9.17)	81 (7.30)	4 (12.90)	
0 levels/GCSEs or CSEs or equivalent	3141 (23.45)	1438 (22.00)	280 (20.66)	2 (22.22)		998 (27.16)	263 (23.69)	7 (22.58)	
NVQ or HND or HNC or equivalent	1152 (8.60)	715 (10.94)	114 (8.41)	0		220 (5.99)	60 (5.41)	3 (9.68)	
None of the above	5141 (38.39)	2292 (35.06)	606 (44.72)	5 (55.56)		1385 (37.70)	530 (47.75)	11 (35.48)	
Household income					<.001				<.001
< £18 K	4478 (33.44)	1962 (30.01)	576 (42.51)	4 (44.44)		1187 (32.31)	453 (40.81)	12 (38.71)	
£18 K ‐ < £52 K	4998 (37.32)	2765 (42.30)	432 (31.88)	4 (44.44)		1330 (36.20)	274 (24.68)	8 (25.81)	
£52 K ‐ < £100 K	1089 (8.13)	708 (10.83)	63 (4.65)	0		253 (6.89)	36 (3.24)	0	
> £100 K	211 (1.58)	144 (2.20)	13 (0.96)	0		44 (1.20)	2 (0.18)	0	
NK	2616 (19.53)	958 (14.66)	271 (20.00)	1 (11.11)		860 (23.41)	345 (31.08)	11 (35.48)	
Smoking status					.016				.004
Never	5800 (43.31)	2368 (36.22)	520 (38.38)	1 (11.11)		1980 (53.89)	646 (58.20)	19 (61.29)	
Previous	5938 (44.34)	3320 (50.79)	634 (46.79)	5 (55.56)		1323 (36.01)	351 (31.62)	5 (16.13)	
Current	1654 (12.35)	849 (12.99)	201 (14.83)	3 (33.33)		371 (10.10)	113 (10.18)	7 (22.58)	
Alcohol consumption					< .001				< .001
Never	1230 (9.18)	321 (4.91)	126 (9.30)	1 (11.11)		475 (13.93)	228 (20.54)	10 (32.26)	
Previous	1154 (8.62)	454 (6.95)	160 (11.81)	1 (11.11)		283 (7.70)	142 (12.79)	2 (6.45)	
Occasionally	4336 (32.38)	1674 (25.61)	361 (26.64)	1 (11.11)		1643 (44.72)	446 (40.18)	9 (29.03)	
Weekly	4813 (35.94)	2876 (44.00)	491 (36.24)	4 (44.44)		1008 (27.44)	225 (20.27)	8 (25.81)	
Daily	1765 (13.18)	1184 (18.11)	198 (14.61)	2 (22.22)		242 (6.59)	53 (4.77)	2 (6.45)	
NK	94 (0.70)	28 (0.43)	19 (1.40)	0		23 (0.63)	16 (1.44)		
Sleep chronotype					.358				<.001
Morning	7092 (52.96)	3469 (53.07)	716 (52.84)	4 (44.44)		2002 (54.49)	534 (48.11)	17 (54.84)	
Evening	4620 (34.50)	2170 (33.20)	436 (32.18)	5 (55.56)		1337 (36.39)	432 (38.92)	13 (41.94)	
NK	1680 (12.54)	898 (13.74)	203 (14.98)	0		335 (9.12)	144 (12.97)	1 (3.23)	
Hypertension	9663 (72.16)	4727 (72.31)	1040 (76.75)	7 (77.78)	.003	2501 (68.07)	824 (74.23)	16 (51.61)	<.001
Insulin use	604 (4.51)	442 (6.76)	122 (9.00)	0	.010	0	0	0	‐
Duration of diabetes (years)	5.15 ± 4.69	5.10 ± 4.65	5.50 ± 5.05	6.94 ± 3.95	.008	5.06 ± 4.66	5.14 ± 4.66	5.98 ± 6.34	.496
Physical activity (MET‐min/week)	2107.58 ± 2143.31	2230.39 ± 2311.45	1845.28 ± 1927.58	743.18 ± 686.99	< .001	2132.33 ± 2036.83	1862.18 ± 1740.39	1838.06 ± 1544.81	<.001
Oily fish intake (portion/week)	1.14 ± 1.12	1.14 ± 1.11	1.14 ± 1.21	1.50 ± 1.17	.635	1.16 ± 1.10	1.10 ± 1.08	1.12 ± 1.11	.382
Vegetable and fruit intake (portion/day)	8.57 ± 4.95	8.26 ± 4.76	8.56 ± 6.06	6.95 ± 4.16	.095	8.96 ± 4.81	9.13 ± 5.17	8.43 ± 3.22	.481
Red meat intake (portion/week)	2.37 ± 1.63	2.47 ± 1.65	2.47 ± 1.90	1.39 ± 0.74	.157	2.18 ± 1.50	2.17 ± 1.55	1.80 ± 1.45	.369
Processed meat intake (portion/week)	1.72 ± 1.48	1.97 ± 1.52	1.88 ± 1.60	1.67 ± 1.30	.144	1.32 ± 1.27	1.36 ± 1.33	0.81 ± 0.97	.047

*Note*: 676 participants lacked handgrip strength or muscle mass data to participate in the sarcopenia classification. Data are presented as mean ± SD for continuous variables or *n* (%) for categorial variables.

Abbreviations: A/AS, advanced; BMI, body mass index; CSE, Certificate of Secondary Education; GCSE, General Certificate of Secondary Education; HNC, Higher National Certificate; HND, Higher National Diploma; MET, metabolic equivalents; NK, not known; NVQ, National Vocational Qualification; O, ordinary.

**FIGURE 1:**
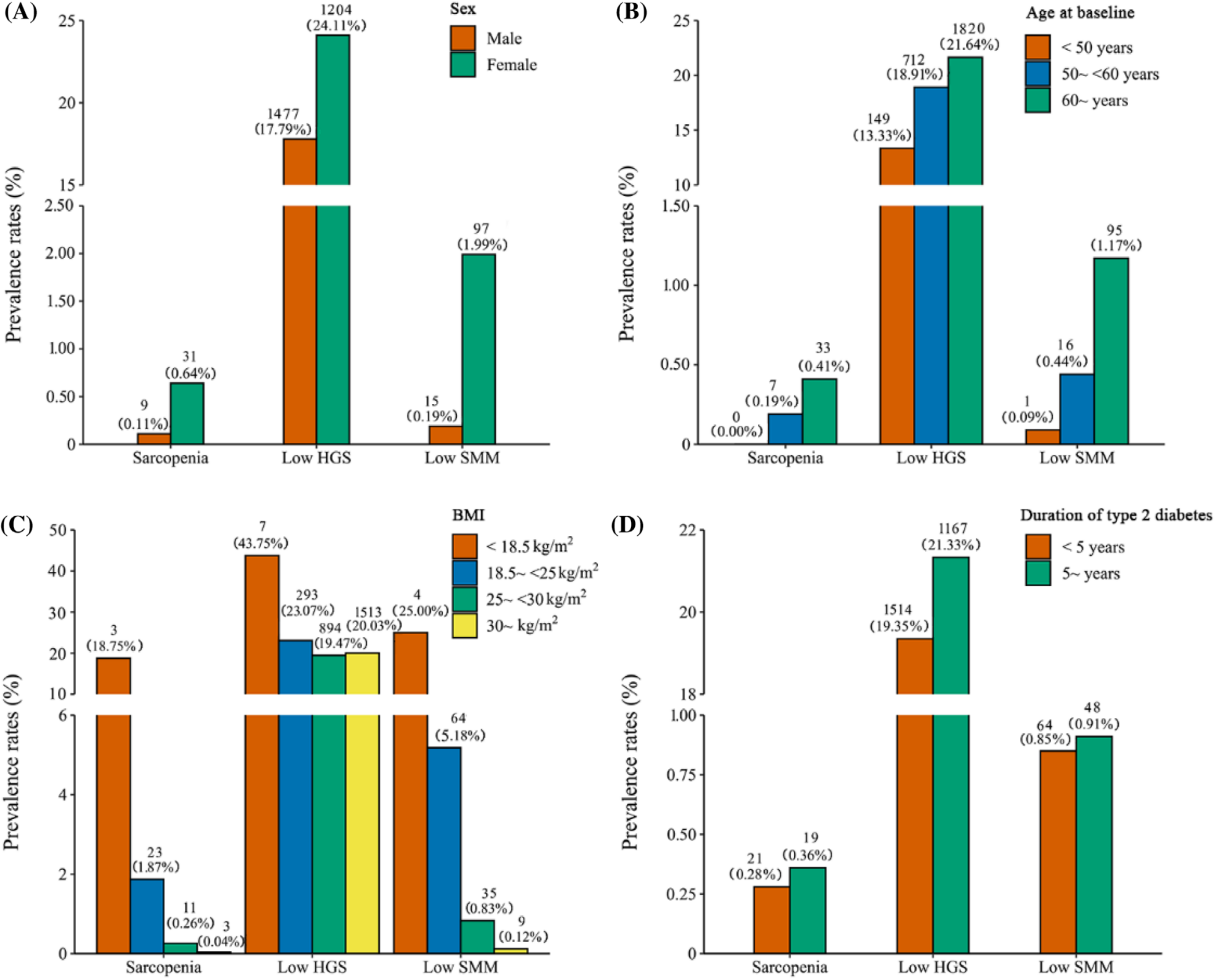
Prevalence of sarcopenia, low handgrip strength and low skeletal muscle mass in populations with different characteristics. (A) Sex. (B) Age. (C) BMI. (D) Duration of type 2 diabetes. BMI, body mass index; HGS, handgrip strength; SMM skeletal muscle mass.

The distribution of sarcopenia, low HGS, and low SMM in the population with different characteristics is presented in Figure 1. In the total population, the prevalence of sarcopenia and low SMM by the EWGSOP2 criteria was low at 0.31% and 0.88%, respectively, whereas the prevalence of low HGS was 21.08%. Nevertheless, the distribution of sarcopenia, low HGS, and low SMM was generally consistent across sex, age, BMI, and duration of type 2 diabetes, showing a higher prevalence in women and an increasing prevalence with age and disease duration, but a decreasing prevalence with increasing BMI. Person correlation analysis showed that there was a positive correlation between HGS and SMM/height^2^ (correlation coefficient: 0.54; *p* < .001).

The association of sarcopenia, low HGS, and low SMM defined according to the EWGSOP2 criteria with all‐cause and CVD mortality is presented in Table 2. Among men, those with sarcopenia had an increased risk for all‐cause mortality compared with those without sarcopenia, with an HR (95% CI) of 2.62 (1.08–6.38), but sarcopenia was not found to be associated with CVD mortality. In women, sarcopenia was not found to be associated with all‐cause mortality or CVD mortality. In sensitivity analyses, we found no statistically significant association between sarcopenia and all‐cause mortality or CVD mortality in either sex after excluding those who died within the first 2 years of follow‐up or non‐White ethnicity (Table S2). When defined according to the FNIH criteria, the risk for all‐cause mortality was increased in the sarcopenia group in men and women, and the risk for CVD mortality was also increased in men (Table S3).

**TABLE 2:** Association between categories of physical capability markers based on EWGSOP2 and all‐cause and CVD mortality in participants with diabetes.

	All‐cause mortality			CVD mortality		
	No. of incidence/participants	Model 1 HR (95% CI)	Model 2 HR (95% CI)	No. of incidence/participants	Model 1 HR (95% CI)	Model 2 HR (95% CI)
Men						
Sarcopenia	1927/7901			497/7901		
Nonsarcopenia	1433/6537	Ref	Ref	358/6537	Ref	Ref
Presarcopenia	489/1355	1.73 (1.56–1.92)	1.59 (1.43–1.77)	137/1355	1.95 (1.60–2.37)	1.68 (1.37–2.06)
Sarcopenia	5/9	3.42 (1.42–8.24)	2.62 (1.08–6.38)	2/9	6.22 (1.54–25.09)	3.12 (0.76–12.91)
Handgrip strength	2124/8301			573/8301		
Normal	1559/6824	Ref	Ref	408/6824	Ref	Ref
Low	565/1477	1.80 (1.63–1.98)	1.62 (1.47–1.79)	165/1477	2.00 (1.67–2.40)	1.71 (1.42–2.06)
Skeletal muscle mass	1944/7949			500/7949		
Normal	1936/7934	Ref	Ref	497/7934	Ref	Ref
Low	8/15	2. 89 (1.44–5.81)	2.43 (1.20–4.93)	3/15	4.83 (1.54–15.15)	3.08 (0.96–9.88)
Women						
Sarcopenia	789/4815			154/4815		
Nonsarcopenia	544/3674	Ref	Ref	101/3674	Ref	Ref
Presarcopenia	237/1110	1.39 (1.19–1.62)	1.33 (1.13–1.55)	52/1110	1.66 (1.16–2.28)	1.37 (0.97–1.93)
Sarcopenia	8/31	1.83 (0.91–3.71)	1.73 (0.85–3.55)	1/31	1.12 (0.15–8.09)	0.84 (0.11–6.26)
Handgrip strength	849/4994			166/4994		
Normal	578/3790	Ref	Ref	107/3790	Ref	Ref
Low	271/1204	1.43 (1.24–1.66)	1.35 (1.17–1.57)	59/1204	1.66 (1.21–2.29)	1.42 (1.02–1.96)
Skeletal muscle mass	805/4863			158/4863		
Normal	781/4766	Ref	Ref	152/4766	Ref	Ref
Low	24/97	1.52 (1.00–2.31)	1.47 (0.96–2.24)	6/97	1.88 (0.81–4.34)	1.85 (0.78–4.39)

*Note*: Model 1 was adjusted age (continuous), BMI (continuous); Model 2 was adjusted for Model 1 plus ethnicity, professional qualifications, gross income, duration of diabetes, history of recent medication for diabetes (insulin), baseline prevalence of hypertension, smoking status, alcohol intake frequency, physical activity, sleep chronotype, and dietary intake (oily fish, fruits and vegetables, red meat, and processed meat).

Abbreviations: BMI, body mass index; CI, confidence interval; CVD, cardiovascular disease; EWGSOP2, European Working Sarcopenia in Older People 2019 recommendations; HR, hazard ratio.

Low HGS was associated with an increased risk of all‐cause mortality and CVD mortality compared with normal HGS. For the classification of SMM, we found an association of low SMM with an increased risk of all‐cause mortality only in men, but not with CVD mortality (Table 2). Stratified analyses showed an increased risk of all‐cause mortality at BMI ≥30 kg/m^2^ and an increased CVD mortality risk at BMI < 30 kg/m^2^ among female patients in the low HGS group (Tables S4 and S5).

Table 3 shows associations between quartiles and continuous variables of HGS and SMM/height^2^ with mortality risk. The risk for all‐cause mortality and CVD mortality among both men and women increased progressively with decreasing HGS quartiles (*p* trend <.05). Using HGS as a continuous variable, a 1 SD decrease in HGS was found to increase the risk for all‐cause mortality by 31% (95% CI: 1.24–1.38) in men and 26% (95% CI: 1.11–1.42) in women; the risk for CVD mortality increased by 35% (95% CI: 1.22–1.50) in men and 43% (95% CI: 1.09–1.89) in women. There was no statistically significant trend association between SMM/height^2^ and mortality risk when it was quartile divided or as a continuous variable. However, when the third quartile (Q3) of SMM/height^2^ was used as a reference, an increased risk for mortality was found in men for Q1 and in women for both Q1 and Q4 (Table S6). In addition, sensitivity analysis showed no significant change in the results of SMM/height^2^ quartiles after further adjusting for fat mass (Table S7).

**TABLE 3:** Association between handgrip strength and muscle mass and all‐cause and CVD mortality in participants with diabetes.

	All‐cause mortality			CVD mortality		
	No. of incidence/participants	Model 1 HR (95% CI)	Model 2 HR (95% CI)	No. of incidence/participants	Model 1 HR (95% CI)	Model 2 HR (95% CI)
Men						
Handgrip strength	2124/8301			573/8301		
Quartile 4	346/1908	Ref	Ref	87/1908	Ref	Ref
Quartile 3	480/2123	1.25 (1.09–1.43)	1.20 (1.04–1.38)	117/2123	1.21 (0.92–1.60)	1.12 (0.85–1.48)
Quartile 2	556/2148	1.41 (1.24–1.62)	1.31 (1.14–1.50)	147/2148	1.48 (1.14–1.93)	1.31 (1.00–1.71)
Quartile 1	742/2122	2.10 (1.84–2.38)	1.86 (1.60–2.08)	222/2122	2.48 (1.94–3.18)	1.99 (1.54–2.57)
*p* for trend		<.001	<.001		<.001	<.001
HR per 1 SD lower HGS	2124/8301	1.41 (1.34–1.48)	1.31 (1.24–1.38)	573/8301	1.51 (1.37–1.67)	1.35 (1.22–1.50)
SMM/height^2^	1944/7949			500/7949		
Quartile 4	572/1987	Ref	Ref	169/1987	Ref	Ref
Quartile 3	449/1988	0.82 (0.72–0.93)	0.82 (0.72–0.94)	106/1988	0.68 (0.53–0.89)	0.70 (0.54–0.91)
Quartile 2	442/1986	0.85 (0.74–0.98)	0.83 (0.72–0.96)	107/1986	0.74 (0.56–0.98)	0.72 (0.55–0.96)
Quartile 1	481/1988	0.98 (0.83–1.14)	0.92 (0.78–1.07)	118/1988	0.88 (0.65–1.20)	0.81 (0.59–1.10)
*p* for trend		.666	.211		.332	.123
HR per 1 SD lower SMM/height^2^	1944/7949	1.12 (1.02–1.23)	1.03 (0.94–1.13)	500/7949	1.11 (0.93–1.32)	0.98 (0.82–1.17)
Women						
Handgrip strength	849/4994			166/4994		
Quartile 4	164/1183	Ref	Ref	28/1183	Ref	Ref
Quartile 3	194/1266	1.07 (0.87–1.32)	1.04 (0.84–1.28)	30/1266	0.96 (0.57–1.60)	0.92 (0.55–1.55)
Quartile 2	212/1270	1.15 (0.94–1.41)	1.06 (0.87–1.31)	48/1270	1.50 (0.94–2.40)	1.36 (0.84–2.17)
Quartile 1	279/1275	1.54 (1.27–1.86)	1.40 (1.15–1.71)	60/1275	1.90 (1.22–2.98)	1.59 (1.00–2.52)
*p* for trend		<.001	<.001		<.001	.023
HR per 1 SD lower HGS	849/4994	1.36 (1.21–1.53)	1.26 (1.11–1.42)	166/4994	1.69 (1.29–2.20)	1.43 (1.09–1.89)
SMM/height^2^	805/4863			158/4863		
Quartile 4	244/1217	Ref	Ref	49/1217	Ref	Ref
Quartile 3	173/1215	0.73 (0.59–0.90)	0.73 (0.59–0.90)	27/1215	0.57 (0.34–0.93)	0.56 (0.34–0.93)
Quartile 2	172/1216	0.73 (0.58–0.91)	0.73 (0.58–0.92)	36/1216	0.76 (0.46–1.26)	0.76 (0.46–1.26)
Quartile 1	216/1215	0.96 (0.76–1.23)	0.94 (0.73–1.20)	46/1215	1.02 (0.59–1.76)	1.02 (0.59–1.76)
*p* for trend		.506	.370		.974	.965
HR per 1 SD lower SMM/height^2^	805/4863	0.90 (0.75–1.07)	0.86 (0.72–1.02)	158/4863	1.01 (0.67–1.54)	0.92 (0.63–1.35)

*Note*: Model 1 was adjusted age (continuous), BMI (continuous); Model 2 was adjusted for Model 1 plus ethnicity, professional qualifications, gross income, duration of diabetes, history of recent medication for diabetes (insulin), baseline prevalence of hypertension, smoking status, alcohol intake frequency, physical activity, sleep chronotype, and dietary intake (oily fish, fruits and vegetables, red meat, and processed meat).

Abbreviations: BMI, body mass index; CI, confidence interval; CVD, cardiovascular disease; HGS, handgrip strength; HR, hazard ratio; SMM, skeletal muscle mass.

**FIGURE 2:**
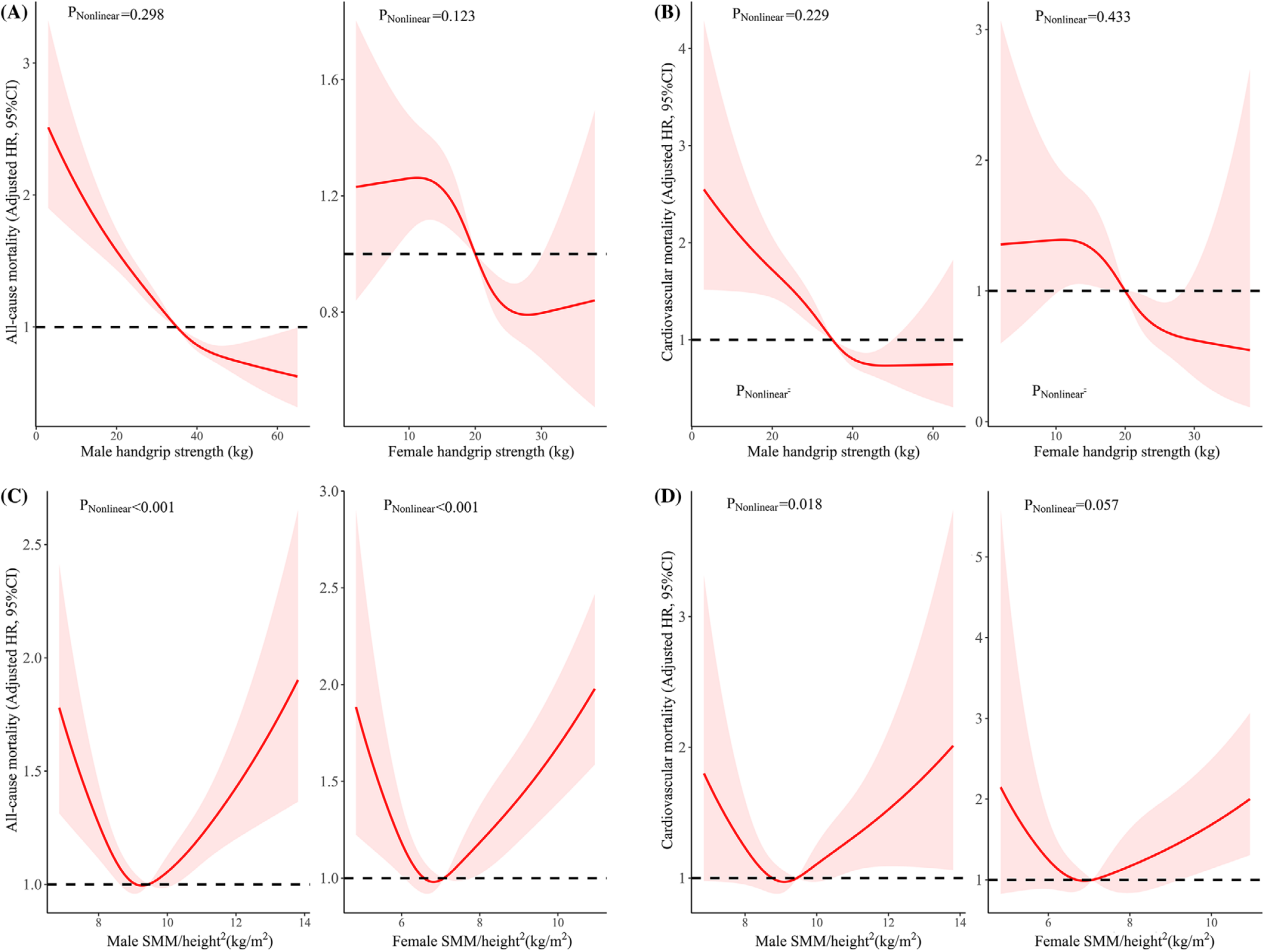
Adjusted hazard ratios (HRs) of mortality using restricted cubic splines. (A) Handgrip strength and all‐cause mortality. (B) Handgrip strength and cardiovascular mortality. (C) Skeletal muscle mass (SMM) and all‐cause mortality. (D) Skeletal muscle mass and cardiovascular mortality. CI, confidence interval.

Nonlinear associations between the exposures (HGS and SMM/height^2^) and mortality risk are presented in Figure 2. There was no nonlinear evidence between HGS and the risk for all‐cause mortality or CVD mortality in both men and women (nonlinear *p* values >.05), but there was significant evidence for a relationship between SMM/height^2^ and all‐cause mortality (nonlinear *p* value <.001). There was a nonlinear association between SMM/height^2^ and the risk of CVD mortality in men (nonlinear *p* value = .018) and a marginal association in women (nonlinear *p* value = .057).

## 4 DISCUSSION

Our prospective study of 13 392 participants with type 2 diabetes in the UK Biobank showed the following results. First, the prevalence of sarcopenia, low HGS, and low SMM was higher in women and in patients with long‐duration diabetes and increased with increasing age and decreasing BMI. Second, very few subjects were defined as having sarcopenia or low SMM according to the EWGSOP2 criteria, and their association with mortality risk was not significant. Low HGS was a better predictor of all‐cause and CVD mortality risk. Third, HGS showed a linear decreasing trend with all‐cause and CVD mortality risk, whereas SMM/height^2^ showed a U‐shaped nonlinear relationship.

The distribution of sarcopenia, low HGS, and low SMM was consistent across sex, age, BMI, and diabetes duration, although the prevalence size varied considerably. In general, the prevalence of sarcopenia was higher in women and in those with a longer duration of type 2 diabetes, and the prevalence increased progressively with age, which was consistent with the results of previous studies.^27^, ^28^, ^29^ However, as BMI decreases, we found an increasing prevalence of sarcopenia, which may also explain the lack of health outcome benefits associated with high SMM.^30^ However, some studies have shown that sarcopenia occurs more frequently in men.^5^, ^31^, ^32^, ^33^, ^34^ This inconsistency may be associated with the age of the included subjects, the presence or absence of type 2 diabetes, the definition of sarcopenia, or the different methods of physical measurement. Previous studies have shown a higher prevalence of sarcopenia in women than in men in populations aged younger than 75 years, whereas in the pooled 85‐year‐old population, the results were reversed.^35^ The populations included in our study were all <75 years. This association, which varies by age and sex, may be related to insulin‐like growth factor‐1 (IGF‐1) levels. Among those in their 70s, men had higher IGF‐1 levels than women, but among those >85 years, men had lower IGF‐1 levels than women.^36^ IGF‐1 is involved in many anabolic pathways in skeletal muscle, and animal trials have shown a protective effect of IGF‐1 against age‐related muscle mass and strength loss.^37^

There is a lack of a uniform definition for the diagnosis of sarcopenia. The EWGSOP2 and FNIH are currently the more used criteria. Our study showed that the number of subjects with sarcopenia or low SMM as determined by the EWGSOP2 criteria was small and sarcopenia was not found to be associated with mortality risk in women. When sarcopenia was defined by the FNIH, the number of patients with sarcopenia increased considerably (641 vs 40), whereas sarcopenia was found to be associated with an increased risk of all‐cause mortality in women and CVD mortality in men. Previous studies have also shown^38^ that there is poor agreement between these two diagnostic criteria for sarcopenia and that the prevalence of diabetes is higher in patients with sarcopenia using the FNIH definition than in the nonsarcopenic group, with the opposite result when using the EWGSOP2 criteria,^39^, ^40^ suggesting that the EWGSOP2 is less sensitive in diabetic patients. Previous studies have also shown that the prevalence of sarcopenia in the type 2 diabetes population is not low, and the use of the EWGSOP2 criteria for its definition may considerably underestimate the impact of sarcopenia.^41^, ^42^, ^43^ The results of a meta‐analysis showed that sarcopenia is associated with a higher risk of mortality, suggesting the need for early screening and diagnosis.^44^ As the indicators and cutoff points for assessing HGS and physical performance are similar, these two criteria identify different sarcopenic populations mainly in the different definitions of low SMM. Considering the difficulty and poor accuracy of current muscle mass measurement methods, muscle strength may be more appropriate as a screening indicator. In many revised guidelines for sarcopenia, muscle strength is also ranked first, because it is recognized that it is superior to muscle mass in predicting poor health outcomes.^1^, ^45^, ^46^ This is consistent with our results, where we found that low HGS was associated with an increased risk of all‐cause mortality and CVD mortality in both men and women, whereas low SMM was not significantly associated with the risk of mortality.

In the diabetic population, the relationship between all‐cause mortality and CVD mortality risk displayed a linear decrease with increasing HGS and a U‐shaped association with increasing SMM/height^2^. Patricio Lopez‐Jaramillo et al^14^ showed a significant negative association between age‐adjusted grip strength (per 1 kg increase) and all‐cause and CVD mortality in both men and women in a diabetic population, consistent with the present study. A meta‐analysis that included data from approximately 2 million men and women^47^ showed that in the general population, higher grip strength levels were associated with a reduced risk of all‐cause mortality (HR = 0.69 [95% CI, 0.64–0.74]) compared with lower grip strength. Previous studies on the relationship between muscle mass and health outcomes in the diabetic population are rare, and a small sample (*n* = 304) retrospective study^16^ showed that all‐cause mortality was higher in patients with low SMM in both men and women. We found a U‐shape between SMM/height^2^ and the risk for all‐cause and CVD mortality, which was consistent with the results of previous studies.^48^, ^49^, ^50^, ^51^, ^52^, ^53^ Overall, either too high or too low muscle mass predicted a higher risk for mortality. Too low implies muscle deficiency, and low muscle mass has been shown to be associated with an increased risk of mortality.^54^, ^55^ Excessive muscle mass may be accompanied by obesity and/or a state of excess body fat that may not be as protective.

To our knowledge, this is the first study to investigate the relationship between grip strength, muscle mass, and sarcopenia simultaneously with all‐cause and CVD mortality in a diabetic population. The strengths of our study are the large sample, being a prospective cohort study, a longer‐term follow‐up, adjustment for as many confounding factors as possible, and the performance of extensive subgroup and sensitivity analyses. However, there are some limitations of our study. First, the small population with sarcopenia and the lack of statistically significant results may be due to the overly strict criteria for the definition of sarcopenia. We further analyzed the relationship between sarcopenia defined by the FINH criteria and outcome, and a relationship between sarcopenia and the risk of mortality was found. Second, in the UK Biobank, 95% of the sample was from White ethnicity, and participants tended to be healthier and wealthier than the national average, thus we are unsure whether the findings are applicable to other populations. Third, SMM was determined by the bioimpedance analysis method, the reliability of which is influenced by several factors, including body water content and electrode placement. Although bioimpedance is not the gold standard for measuring muscle mass, previous studies have shown that the use of bioimpedance to estimate muscle mass in the UK Biobank population was in good agreement with dual‐energy X‐ray analysis (*r* = 0.868, *p* < .0001).^56^ Fourth, the UK Biobank inclusion population was all aged <73 years, and given the trend of increasing sarcopenia with age, we predict a greater effect in the more advanced age groups. Finally, although adjusted for most confounders, there may be other potentially unmeasured or unknown confounders that could affect the results, such as the total energy intake.

In conclusion, we found that low grip strength seemed to be a better predictor for all‐cause mortality and CVD mortality risk in patients with type 2 diabetes compared to low muscle mass. Grip strength displayed a linear downward trend with mortality risk, whereas muscle mass showed a U‐shaped relationship, and either too low or too high muscle mass was associated with an increased risk of all‐cause and CVD mortality. Screening and diagnosis of sarcopenia in the diabetic population appear necessary, and the focus needs to be on the occurrence of low HGS.

## AUTHOR CONTRIBUTIONS

Lingqi Wei, Jingjing Zeng, and Menglin Fan contributed equally to this work. Shaoyong Xu, Lingqi Wei, Jingjing Zeng, and Menglin Fan conceived the idea for the paper. Shaoyong Xu acquired the data. Jingjing Zeng, Menglin Fan, and Bo Chen prepared the data for analysis and analyzed the data. Shaoyong Xu, Jingjing Zeng, Menglin Fan, Bo Chen, Xiaying Li, and Ying Li interpreted the results. Lingqi Wei, Jingjing Zeng, and Menglin Fan wrote the first draft. All authors critically revised the paper for intellectual content and approved the final version of the manuscript.

## FUNDING INFORMATION

The study was partly supported by the Young Talents Project of Hubei Provincial Health Commission, China (Grand number WJ2021Q012); Science and Technology Research Key Project of Education Department of Hubei Province, China (Grand number D20212602); Sanuo Diabetes Charity Foundation, China; and Xiangyang Science and Technology Plan Project, China (Grand number 2019ZD12).

## DISCLOSURE

The authors declare no competing interests.

## Supporting information


**Figure S1.** Flow chart of study participants.

**Table S1.** Strengthening the Reporting of Observational Studies in Epidemiology (STROBE) checklist.

**Table S2.** Sensitivity analyses for association between sarcopenia and all‐cause and cardiovascular disease (CVD) mortality in participants with diabetes.

**Table S3.** Association between categories of sarcopenia based on Foundation for the National Institutes of Health (FNIH) criterion and all‐cause and cardiovascular disease (CVD) mortality in participants with diabetes.

**Table S4.** Stratification analyses for association between low handgrip strength and all‐cause and cardiovascular disease (CVD) mortality in male participants with diabetes.

**Table S5.** Stratification analyses for association between handgrip strength and all‐cause and cardiovascular disease (CVD) mortality in female participants with diabetes.

**Table S6.** Association between SMM/height^2^ quartiles and all‐cause and cardiovascular disease (CVD) mortality in participants with diabetes.

**Table S7.** Sensitivity analysis for association between skeletal muscle mass (SMM)/height^2^ quartiles and all‐cause and cardiovascular disease (CVD) mortality in participants with diabetes.

## Data Availability

UK Biobank data can be requested by bona fide researchers for approved projects, including replication, through https://www.ukbiobank.ac.uk/.
