# Supplementary material for: Associations between handgrip strength and skeletal muscle mass with all‐cause mortality and cardiovascular mortality in people with type 2 diabetes: A prospective cohort study of the UK Biobank
Source: J Diabetes. 2023 Aug 22;16(1):e13464. doi: 10.1111/1753-0407.13464 (PMC10809293; doi:10.1111/1753-0407.13464)
Supplement: Supplementary file 1 — Figure S1. Flow chart of study participants. [file JDB-16-e13464-s005.pptx]

## Slide 1
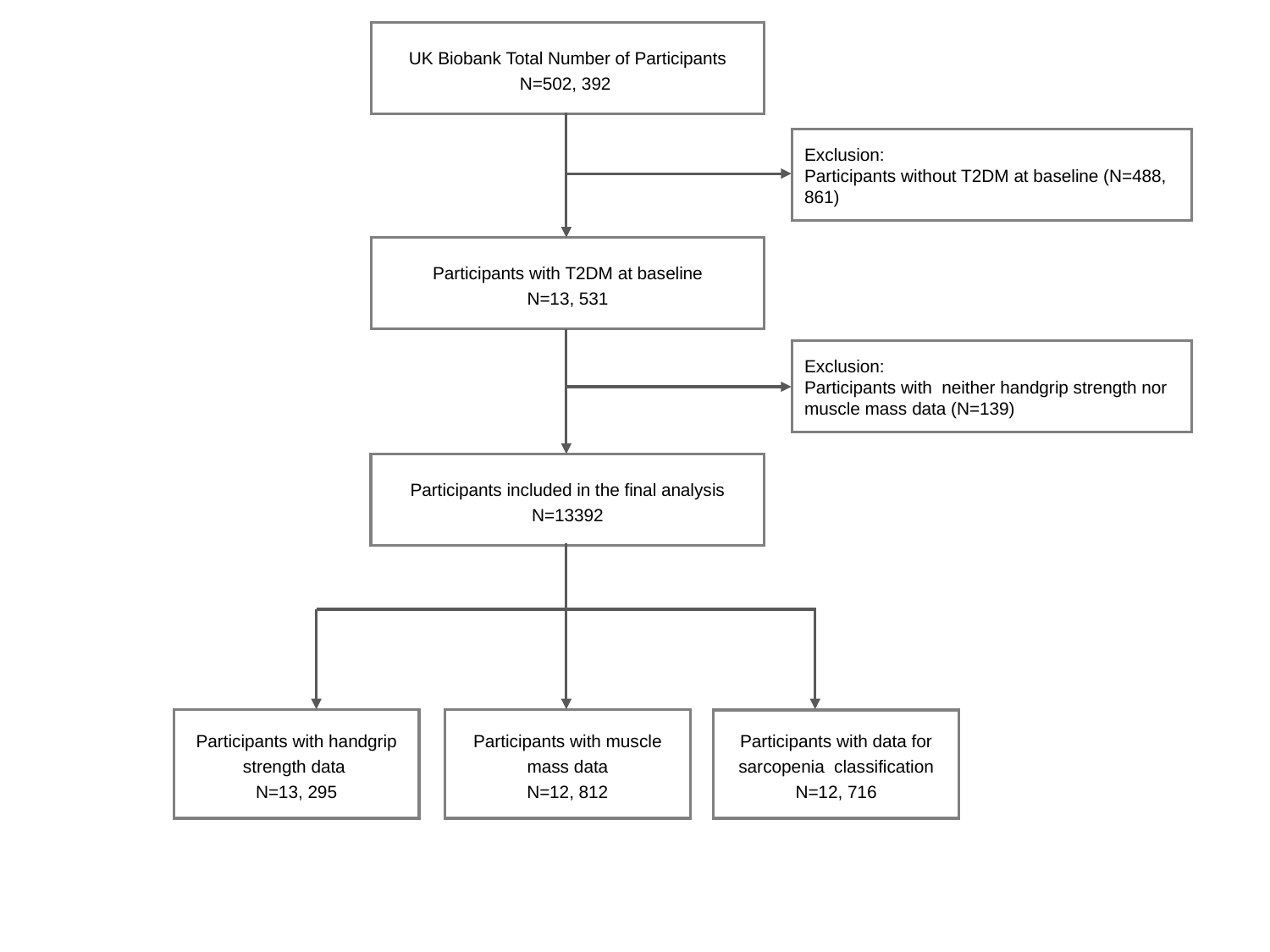

UK Biobank Total Number of Participants
N=502, 392
Exclusion:
Participants without T2DM at baseline (N=488, 861)
Participants with T2DM at baseline
N=13, 531
Exclusion:
Participants with neither handgrip strength nor muscle mass data (N=139)
Participants included in the final analysis
N=13392
Participants with handgrip strength data
N=13, 295
Participants with muscle mass data
N=12, 812
Participants with data for sarcopenia classification
N=12, 716
