# Supplementary material for: Associations between handgrip strength and skeletal muscle mass with all‐cause mortality and cardiovascular mortality in people with type 2 diabetes: A prospective cohort study of the UK Biobank
Source: J Diabetes. 2023 Aug 22;16(1):e13464. doi: 10.1111/1753-0407.13464 (PMC10809293; doi:10.1111/1753-0407.13464)
Supplement: Supplementary file 8 — Table S7. Sensitivity analysis for association between skeletal muscle mass (SMM)/height2 quartiles and all‐cause and cardiovascular disease (CVD) mortality in participants with diabetes. [file JDB-16-e13464-s003.docx]

**Supplementary Table 7 Sensitivity analysis for association between SMM/height^2^ quartiles and all-cause and CVD mortality in participants with diabetes**

|  | **All-cause mortality** | | |  | **CVD mortality** | | |
| --- | --- | --- | --- | --- | --- | --- | --- |
|  | **No. of incidence/ participants** | **Model 1**  **HR (95% CI)** | **Model 2**  **HR (95% CI)** |  | **No. of incidence/ participants** | **Model 1**  **HR (95% CI)** | **Model 2**  **HR (95% CI)** |
| **Men** |  |  |  |  |  |  |  |
| Quartile 4 | 572/1987 | Ref | Ref |  | 169/1987 | Ref | Ref |
| Quartile 3 | 449/1988 | 0.78(0.69-0.88) | 0.81 (0.71-0.92) |  | 106/1988 | 0.68 (0.53-0.89) | 0.67 (0.52-0.87) |
| Quartile 2 | 442/1986 | 0.78 (0.69-0.89) | 0.81 (0.71-0.92) |  | 107/1986 | 0.66 (0.51-0.85) | 0.69 (0.53-0.89) |
| Quartile 1 | 481/1988 | 0.86 (0.75-0.98) | 0.87 (0.76-1.00) |  | 118/1988 | 0.74 (0.57-0.95) | 0.74 (0.57-1.01) |
| **Women** |  |  |  |  |  |  |  |
| **SMM/height^2^** | 805/4863 |  |  |  | 158/4863 |  |  |
| Quartile 4 | 244/1217 | Ref | Ref |  | 49/1217 | Ref | Ref |
| Quartile 3 | 173/1215 | 0.69 (0.57-0.85) | 0.71 (0.58-0.87) |  | 27/1215 | 0.52 (0.32-0.84) | 0.54(0.33-0.87) |
| Quartile 2 | 172/1216 | 0.68 (0.55-0.83) | 0.70 (0.57-0.87) |  | 36/1216 | 0.66 (0.42-1.05) | 0.71 (0.45-1.13) |
| Quartile 1 | 216/1215 | 0.87 (0.71-1.08) | 0.89 (0.72-1.11) |  | 46/1215 | 0.84 (0.53-1.34) | 0.92 (0.57-1.47) |

CVD, cardiovascular disease; HR, hazard ratio; CI: confidence interval; SMM, Skeletal muscle mass; Model 1 was adjusted age (continuous), fat mass (continuous); Model 2 was adjusted for Model 1 plus ethnicity, professional qualifications, gross income, duration of diabetes, history of recent medication for diabetes (insulin), baseline prevalence of hypertension, smoking status, alcohol intake frequency, physical activity, sleep chronotype, and dietary intake (oily fish, fruits and vegetables, red meat, and processed meat).
